# Chromosome-Level Assembly of Male *Opsariichthys bidens* Genome Provides Insights into the Regulation of the GnRH Signaling Pathway and Genome Evolution

**DOI:** 10.3390/biology11101500

**Published:** 2022-10-13

**Authors:** Dong Liu, Lang Gui, Yefei Zhu, Cong Xu, Wenzong Zhou, Mingyou Li

**Affiliations:** 1 Key Laboratory of Integrated Rice-Fish Farming, Ministry of Agriculture and Rural Affairs, Shanghai Ocean University, Shanghai 201306, China; 2 Shanghai Universities Key Laboratory of Marine Animal Taxonomy and Evolution, Shanghai Ocean University, Shanghai 201306, China; 3 Key Laboratory of Exploration and Utilization of Aquatic Genetic Resources, Ministry of Education, Shanghai Ocean University, Shanghai 201306, China; 4 Key Laboratory of Integrated Rice-Fish Farming, Ministry of Agriculture and Rural Affairs, Eco-Environmental Protection Research Institute, Shanghai Academy of Agricultural Sciences, 1000 Jinqi Road, Shanghai 201403, China

**Keywords:** Cyprinid fish, hook snout carp, sexual dimorphism, comparative genomics, GnRH signaling

## Abstract

**Simple Summary:**

The important farmed fish *Opsariichthys bidens* exhibits sexual dimorphism in growth, with males growing significantly faster than females. However, the mechanism underlying the complex traits is still unknown. According to the assembled genome of the male *O. bidens* in this study, we found that 78 expanded genes were involved in the GnRH signaling pathway, regulating the synthesis and secretion of luteinizing hormone and glycoprotein hormones, further acting on male growth by inducing growth hormone. Compared to the released female genome, the male chromosome LG06 showed over 97% similarity to the female’s GH14/GH38. The LG06 harbored male-specific genes pointing to a centric fusion of the acrocentric chromosomes GH14 and GH38. Further compared to the genome of grass carp, we found that chromosomal diversity resulted from ancestral chromosome breakage. Our results provide a valuable genetic resource for the investigation of sex-determining mechanisms, regulating sexual dimorphism, and generating molecular-guided breeding programs for *O. bidens*.

**Abstract:**

The hook snout carp *Opsariichthys bidens* is an important farmed fish in East Asia that shows sexual dimorphism in growth, with males growing faster and larger than females. To understand these complex traits and improve molecular breeding, chromosome-level genome assembly of male *O. bidens* was performed using Illumina, Nanopore, and Hi-C sequencing. The 992.9 Mb genome sequences with a contig N50 of 5.2 Mb were anchored to 38 chromosomes corresponding to male karyotypes. Of 30,922 functionally annotated genes, 97.5% of BUSCO genes were completely detected. Genome evolution analysis showed that the expanded and contracted gene families in the male *O. bidens* genome were enriched in 76 KEGG pathways, and 78 expanded genes were involved in the GnRH signaling pathway that regulates the synthesis and secretion of luteinizing hormone and glycoprotein hormones, further acting on male growth by inducing growth hormone. Compared to the released female *O. bidens* genome, the number of annotated genes in males was much higher (23,992). The male chromosome LG06 exhibited over 97% identity with the female GH14/GH38. Male-specific genes were identified for LG06, where structural variation, including deletions and insertions, occurred at a lower rate, suggesting a centric fusion of acrocentric chromosomes GH14 and GH38. The genome-synteny analysis uncovered significant inter-chromosome conservation between male *O. bidens* and grass carp, the former originating from ancestral chromosome breakage to increase the chromosome number. Our results provide a valuable genetic resource for studying the regulation of sexual dimorphism, sex-determining mechanisms, and molecular-guided breeding of *O. bidens*.

## 1. Introduction

The hook snout carp *Opsariichthys bidens* Günther, an endemic Asiatic Cyprinid with high economic value, has become a commercially emerging aquaculture fish. *O. bidens* inhabits the tributaries of rivers and prefers to occupy mountainous streams. It has been cultured in large mountain communities and has significantly increased farmer income in the past two decades [1,2]. The hook snout carp is a small to medium-sized fish distributed across China’s main drainages, including the Jiulongjiang, Huaihe, Yellow, Yangtze, and Pearl rivers, and expanding to Hainan drainages in China, Korea, Japan, and Vietnam [3]. The long-distance dispersal distribution of hook snout carp was driven by the uplift of the Qinghai–Tibet Plateau and Pleistocene glacial cycles, causing morphological variations, such as lateral line scales and populations with particular shapes [3]. Genetic diversity examined via mitochondria-based phylogeny has revealed that hook snout carp are clustered into five different evolutionary lineages [4], revealing potential candidates for fishery resources for hook snout carp breeding in the future.

*O. bidens* exhibits sexual dimorphism in growth, with male individuals showing faster growth than females, possibly because of the sexually different systems [5]. Sex-related genes, such as *dazl*, *dnd*, *nanog*, *piwi*, *vasa*, *zp4*, *amh*, *cyp19a*, and *dmrt1*, were identified in hook snout carp using transcription analysis [6]. In addition, sex-biased miRNAs of *O. bidens* were investigated [7]. Furthermore, most recently, a long-term-cultured spermatogonia stem cell line that could produce sperm in vitro was established in *O. bidens* [8]. Regarding the morphological traits of hook snout carp, female individuals have plain bodies, while male individuals have irregular dark bars on the side of their bodies [9,10]. During the reproductive season, male individuals exhibit nuptial coloration on the sides of the body and the surfaces of the head, pectoral, and extended-anal fins with nuptial organs, which facilitates matching with females and improves fertilization and reproductive success rates [9]. This reproductive strategy drives chromosome evolution by altering the strength of sexually antagonistic selection [11]. However, the presumed sex-related chromosomes of hook snout carp have not been previously identified because of the absence of a fully sequenced genome, which makes it difficult to elucidate the sexual dimorphism mechanism of hook snout carp.

High-quality genome assembly at the chromosome level would be helpful for aquaculture breeding and genetic research. The available genomes were used as references for the genetic architecture of morphological characters and dig-specific markers to help molecular breeding with important traits [12]. The whole genomes of economically important aquaculture fishes, including the common carp *Cyprinus carpio* Linnaeus [13], half-smooth tongue sole *Cynoglossus semilaevis* [14], grass carp *Ctenopharyngodon idella* (Valenciennes) [15], Japanese flounder *Paralichthys olivaceus* [16], yellow catfish *Pelteobagrus fulvidraco* [17], bighead carp *Hypophthalmichthys nobilis* (Richardson) [18], and Gibel carp/Prussian carp *Carassius gibelio* [19], have been released. Genetic analyses of complex traits, including sex control, disease resistance, hypoxia tolerance, and efficient feed utilization, have been widely performed, and breeding of a new aquaculture variety is being conducted in fisheries, which greatly relies on genome data [20].

Although the female hook snout carp genome was presented in data format [10], its annotation profile is still lacking. It is limited to serving as a reference for finely mapping and characterizing quantitative trait loci, sex-linked DNA markers, sex control, and genetic breeding of hook snout carp. The female hook snout carp genome is 814.71 Mb and anchored to 39 chromosomes (2n = 78), the largest number in the Danioninae subfamily [10,21]. The diploid chromosome number of hook snout carp varies in different populations: 74 for Sichuan in Yangtze River drainages, 76 for Guangdong in Pearl River drainages, and 78 for Japan [22]. Female *O. bidens* individuals whose 78-chromosome genome has been sequenced were collected from Zhejiang in the Yangtze River drainages [10]. A few fish species show different chromosome numbers due to differences in sex chromosomes. For example, a rocky reef fish, *Oplegnathus fasciatus* (Temminck and Schlegel), shows 2n = 48 for female individuals and 47 for male individuals because of a centric fusion of male acrocentric chromosomes in the formation of the sex-determining system [23]. Sex chromosomes are responsible for sex regulation and determination [24]. Hook snout carp display a variety of chromosome numbers, providing insight into the evolution of sex-related chromosomes in fish.

Here, we report the chromosome-level genome assembly of male *O. bidens* using Illumina short-read sequencing, Nanopore long-read sequencing, and the Hi-C technique. The assembled genome of 992.9 Mb was anchored to 38 chromosomes, corresponding to our karyotype analysis. In total, 36,738 functional genes were identified. This allowed us to further discover male hormone release via the gonadotropin-releasing hormone (GnRH) pathway, which plays a vital role in developing secondary sex characteristics in *O. bidens*. For the first time, to the best of our knowledge, we identified a reunion of male chromosomes carrying expanded genes involved in the GnRH pathway by synteny-based chromosome comparison between male and female *O. bidens*. In addition, the genome of male *O. bidens* compared to that of *C. idella* showed that the chromosome broke off to increase the chromosome number during the evolutionary history of fish. The male *O. bidens* genome provides valuable genomic data for further studies on conservation genetics, sex-determining mechanisms, and all-male and hypoxia-resistant breeding.

## 2. Materials and Methods

### 2.1. Sample Collection, Sequencing, and Karyotype Analysis

*O. bidens* were collected originally from Jinhua Hengyuan Agricultural Science and Technology Co., Ltd. in Dongyang City, China, and raised in an automated breeder system with a 14 h/10 h light/dark cycle (26 ± 1 °C) at the Shanghai Ocean University Aquaculture Center (Shanghai, China). The study was performed following the Declaration of Helsinki and approved by the Shanghai Ocean University Animal Care and Use Committee with Approval Number SHOU-2021-118 (18 March 2021). One adult male *O. bidens* was used for genome sequencing and assembly. Physiological sex was identified using the squeezing method to produce seminal fluid from the fish abdomen. Muscle tissue was collected and treated following the QIAamp DNA Mini Kit (QIAGEN, Hilden, Germany) protocol and was quickly frozen in liquid nitrogen for genomic DNA (gDNA) sequencing. Tissues including the testis, ovary, brain, heart, liver, kidney, muscle, eyes, and skin of the male individual were subjected to transcriptome sequencing using TRIzol-extracted total RNA from the pooled samples.

The gDNA extracted from the muscle tissue was randomly sheared to 300–500 bp fragments and amplified using PCR. A DNA library with an insert size of 350 bp was prepared and constructed using DNA library prep kits (TruSeq, Illumina Inc., San Diego, CA, USA). The short insert library was sequenced using the Illumina HiSeq 2500 platform in 150 PE mode to estimate genome size and heterozygosity by k-mer frequency distribution using Genomescope [25]. Meanwhile, the gDNA extracted from fresh muscle was used to construct the DNA Nanopore library for long-read sequencing using the PromethION platform (Oxford Nanopore, Oxford, UK), according to the manufacturer’s instructions.

To prepare the Hi-C library for chromosome-level genome assembly, 1 g of muscle tissue from the same male individual was fixed with formaldehyde, digested using HindIII (restriction enzyme), biotin marking, physical shearing, and DNA amplification, and finally used to construct the Hi-C library with insert sizes of 350 bp. As previously described, the Hi-C library was sequenced using the Illumina HiSeq 2500 platform in PE150 mode [26].

Transcriptome sequencing was conducted to estimate the coverage rate of the assembled genome over gene regions and predict gene models. Total RNA was extracted using TRIzol reagent (Invitrogen, Carlsbad, CA, USA) from nine tissue pools collected from the same male individual mentioned above. RNA quality was assessed using a Nanodrop spectrophotometer (Labtech, Ringmer, UK). The RNA was used to construct the Illumina RNA-seq library and sequenced using an Illumina NovaSeq 6000 in PE150 mode (Illumina), as previously described [27].

The karyotype of the hook snout carp was examined based on metaphase spread from the head kidney cells of 6-month-old fish. Chromosome samples were obtained through phytohemagglutinin injection, colchicine air-drying, and Giemsa staining, as previously described [28].

### 2.2. Genome Assembly

Nanopore long reads were first corrected to obtain clean reads using the Canu package [29]. Genome assembly was performed using SMART denovo (https://github.com/ruanjue/smartdenovo) and polished in three runs for error-corrected long reads using the gDNA Illumina short reads by Pilon with default parameters to produce the nanopore-assembled genome of *O. bidens* [30]. Genome integrity was assessed using the gDNA short reads by the underlying aligned rate in BWA [31]. The number of genes in the CEGMA database was presented in the assembly [32] and further used to predict genes in the assembled genome using BUSCO with the Vertebrata-odb10 database [33].

For chromosome assembly, the Hi-C technique was applied. Raw Hi-C sequencing data were filtered to obtain high-quality clean reads using HiC-Pro with default parameters [34], and the clean-read pairs were mapped to the polished *O. bidens* genome using BWA in end-to-end mode [31]. Only valid interaction pairs were used to construct the chromosome-level genome of *O. bidens* using LACHESIS with default parameters [35].

### 2.3. Genome Annotation

Repetitive elements, including transposable elements and simple sequence repeats, were predicted using LTR-FINDER [36] and RepeatScout [37]. Repeat types were classified using PASTEClassifier [38]. The Repbase database [39] was used to scan and identify the predicted repeats in the genome of *O. bidens* based on homology-based alignment using RepeatMasker [40].

Gene structure analysis was performed using three combined methods: de novo prediction, homology-based prediction, and transcriptome-based prediction. De novo analysis was performed using GenScan [35] and Augustus [41]. Genes from the *Danio rerio* and *Ctenopharyngodon idellus* NCBI database, were used for homology-based analysis in Gemoma [42]. Transcript annotation results from nine *O. bidens* tissues were used to perform transcriptome-based prediction using TransDecoder (http://transdecoder.github.io) and GeneMarkS-T [43]. Finally, the three evidence sets were integrated using EVM [44]. The microRNAs and rRNAs were identified using Blastn against the Rfam database [45], and tRNAs were identified using tRNAscan-SE [46].

Gene functional annotations were performed using BLAST with an e-value of 1 × 10^−5^ in searching the NCBI non-redundant protein (NR), EuKaryotic Orthologous Groups (KOG), Gene Ontology (GO), Kyoto Encyclopedia of Genes and Genomes (KEGG) [47], and Tremble databases to identify homologous protein-coding genes [48]. Gene ontology terms were assigned to genes based on the NR annotation information. Gene functional classes were determined based on the KOG database, and functional pathways were analyzed using the KEGG database. Pseudogenes were identified by searching the identified gene sets using GeneWise [49].

### 2.4. Genome Evolution Analysis

The protein sequences of different species downloaded from NCBI, including Sinocyclocheilus rhinocerous, Anabarilius grahami, Labeo rohita, Danio rerio, Oryzias latipes, Takifugu rubripes, Cyprinus carpio, and Gasterosteus aculeatus (Table S1), together with *O. bidens* were analyzed using an all-to-all BLAST search with an e-value of 1e-7 to obtain orthologous genes. These genes were clustered into families to identify species-unique gene families using OrthoMCL [50]. Single-copy orthologous gene clusters were extracted from the OrthoMCL clustering results. Single-copy gene families were used to construct phylogenetic trees based on the Bayes model using PhyML [51]. The divergence time was estimated using the PAML Mcmctree with the JC69 model [52]. Several calibration times were verified using the TimeTree website (http://www.timetree.org).

Gene family size dynamics, including expansion or contraction, were assessed using Cafe v5.0 (San Francisco, CA, USA) based on OrthoMCL’s results and phylogenetic trees [53]. Functional annotations of expanded and contracted genes were performed using BLAST to search the Pfam database [54].

### 2.5. Comparison Analysis of Chromosomes

Synteny relationships between the male and female *O. bidens* genomes were analyzed using MUMmer4 with default settings [55]. The alignment results were filtered for more than 85% identity, and syntenic blocks of chromosomes were visualized using Circos [56]. The female *O. bidens* genome (https://ngdc.cncb.ac.cn/gwh/Assembly/22234; Accession Number: GWHBEIO00000000) was annotated using a homology-based approach to search the *D. rerio* NCBI database using GeMoMa [42]. The annotated protein-coding genes of male and female *O. bidens* genomes were used by Blastp (identity ≥ 95% and e-value ≤ 1 × 10^−5^) to identify the male/female-specific gene sets. Structural variants of *O. bidens* genomes were analyzed using Assemblytics [57].

To uncover the unique chromosomal evolution mechanism of the *O. bidens* genome, synteny comparison of the hook snout carp and grass carp genomes [58] was performed using JCVI with c-scores of >0.7, and tandem-nmax = 10 as filtering values [59].

## 3. Results

### 3.1. Genome Assembly and Integrity Assessment

Using 90.47 Gb Illumina data, the genome size of male *O. bidens* was estimated to be 813.1 Mb based on the 17-kmer peak with a depth of 111.3 × coverage (Figure S1; Table S2). The 99.7 Gb data obtained from Nanopore sequencing indicated a 122.6-fold range of the genome (Table S2). Low-quality reads and adapter sequences were eliminated from the raw data to give 95.1 Gb clean reads for subsequent genome assembly. After the Nanopore clean reads were corrected and polished, 992.9 Mb sequences were obtained for the male *O. bidens* genome, including 1373 contigs with a contig N50 length of 5.2 Mb. The assembled genome size was larger than the estimated one because of a high heterozygosity ratio of 0.58% (Figure S1), as previously described [23]. The GC content of the whole genome was 37.9%. The mapping rate of Illumina short, clean reads with the entire genome was 99.5%. Out of the 458 genes in the eukaryotic genome CEG database, 456 were present in assemblies (99.6%), and 97.5% of BUSCO genes were wholly found in the genome of *O. bidens* using the vertebrata_odb10 database (Table S3). This evidence indicates a complete high-quality genome.

**Figure 1:**
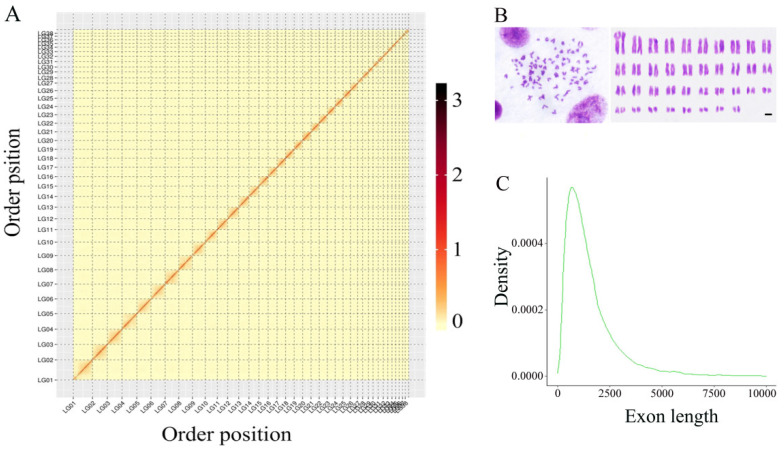
The Hi-C heatmap, karyotypes, and exon length of the male *O. bidens*. (**A**) A Heat map generated by Hi-C technology presented contact matrices of thirty-eight chromosomes. The color bar indicates the logarithm of the strength of the contact density. (**B**) The karyotypes of male *O. bidens*’ genome. (**C**) The distributions of gene exon length in male *O. bidens*’ genome.

Raw reads of 55.96 Gb by Hi-C library sequencing of approximately 68.8 × coverage of the genome were used to construct a chromosome-level assembly (Table S2). A total of 218.09 million read pairs (64.4%) were uniquely mapped to the nanopore draft genome. Finally, 54.15 million read pairs (24.83%) provided valid interaction information for chromosome construction. Using the valid Hi-C information, 1864 contigs (approximately 992.91 Mb) were produced and further clustered into 1461 scaffolds anchored on the 38 chromosomes. The boundaries between different chromosomes were clear, and every chromosome showed strong interactions (Figure 1A), which was consistent with the karyotype of the male hook snout carp (Figure 1B). The contig and scaffold N50 values reached 2.85 Mb and 19.44, respectively (Table S1). The final genome at the chromosome level was 886.81 Mb, representing 89.31% of nanopore genome sequences.

### 3.2. Genome Annotation

About 357.31 Mb, accounting for 43.23% of the assembled genome, was identified as repetitive elements, including transposable elements, SSR, and unknown elements. Class I and II transposable elements accounted for 23.34% and 12.59% of the assembled genome, respectively, and SSR accounted for 0.59% (Table 1).

**Figure 2:**
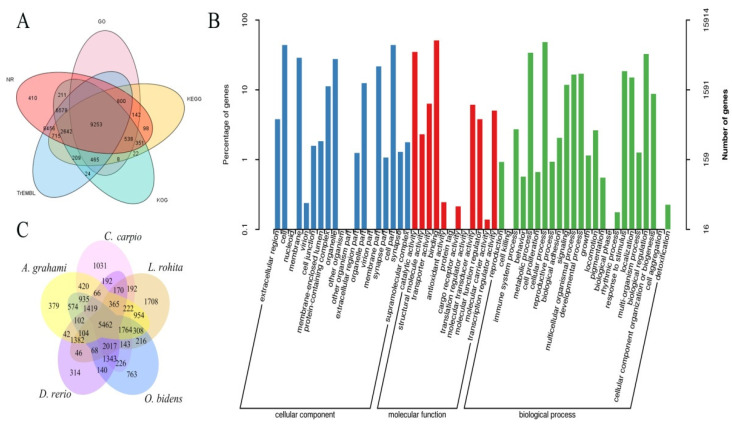
Gene annotation and function of male *O. bidens*’ genome. (**A**) Different database-annotated genes. (**B**) Gene functionally classification by GO analysis. (**C**) A Venn diagram showing the number of species-specific and shared gene orthogroups of *O. bidens* between *D. rerio*, *L. rohita*, *C. carpio*, and *A. graham*.

De novo, homology, and transcript-based methods were used to predict the gene models. The expected results were integrated, and 36,738 non-redundant genes were obtained using the EVM software (Table S4). The average exon length per gene was 2 kb (Figure 1C), the average exon number per gene was 8.11, and the mean CDS length was 1492.64 bp, indicating a relatively fine consistency of the genome assembly. A total of 1829 pseudogenes, 450 miRNAs, 5616 rRNAs, and 3280 tRNAs were annotated in the male *O. bidens* genome (Table 2). A total of 30,922 genes were functionally annotated using the GO, KEGG, KOG, TrEMBL, and NR databases, representing 84.17% of the predicted genes (Figure 2A). According to GO analysis, these annotated genes were functional in cellular components, molecular functions, and biological processes (Figure 2B). Of the annotated genes in the male *O. bidens* genome assembly, 5462 were orthologous to four species, *D. rerio*, *L. rohita*, *C. carpio*, and *A. graham* (Figure 2C).

### 3.3. Genome Phylogeny, Expansion, and Contraction of Gene Families

**Figure 3:**
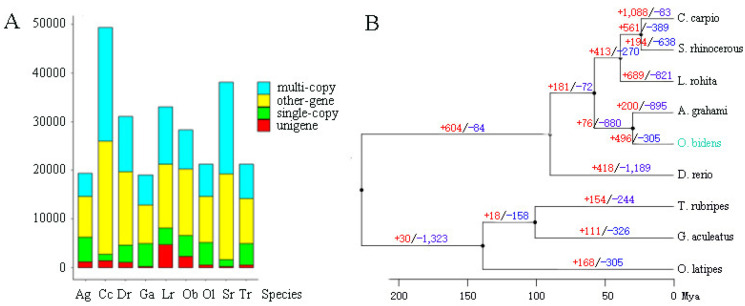
A phylogenetic tree based on single-copy genes from the nine genome sequences. (**A**) The number of orthologous for nine fish species. Names for species are abbreviated to Ag, *Anabarilius grahami*; Cc, *Cyprinus carpio*; Dr, *Danio rerio*; Ga, *Gasterosteus aculeatus*; Lr, *Labeo rohita*; Ob, *Opsariichthys bidens*; Ol, *Oryzias latipes*; Sr, *Sinocyclocheilus rhinocerous*; Tr, *Takifugu rubripes*. (**B**) A bayesian phylogenetic tree, gene families, and divergence time of male *O. bidens*. The bayesian posterior probability and the maximum likelihood bootstrap had support values of 100%. The numbers above the lines indicate gene family expansions (+ and red) and contractions (− and green).

In total, 4350 single-copy orthologs and 18,271 gene families were identified in the assembled genome of male *O. bidens* by clustering homologous genes in *S. rhinocerous*, *A. grahami*, *L. rohita*, *D. rerio*, *O. latipes*, *T. rubripes*, *C. carpio,* and *G. aculeatus* (Figure 3A). A phylogenetic tree was constructed using these single-copy orthologues (Figure 3B). Together with calibration times, the results showed that *O. bidens* was divided from *D. rerio* at approximately 89.69 Mya, separated from *L. rohita* at about 57.77 Mya, then isolated from *A. grahami* at approximately 29.74 Mya, indicating a rapid differentiation among these species.

A total of 496 and 305 gene families were significantly expanded and contracted in the male *O. bidens* genome (*p* < 0.05), respectively (Figure 3B). These gene families were mainly involved in 60 GO terms (Table S5; Figure S2), such as metabolic processes in biological processes, organelles in cellular components, and catalytic activity in molecular functions. The expanded and contracted gene families were enriched in 76 KEGG pathways (Table S6; Figure S3), such as the calcium signaling pathway, ABC transporters, GnRH signaling pathway, melanogenesis, and adrenergic signaling in cardiomyocytes.

### 3.4. Expanded Genes Involved in the GnRH Signaling Pathway

**Figure 4:**
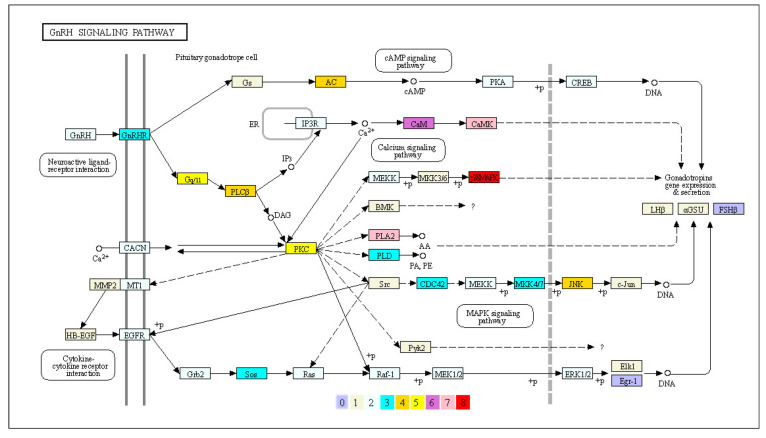
GnRH signaling pathway of male *O. bidens*’ genome. The gene-marked colors indicate the gene copy number presented on this signaling pathway (KEGG ID: ko04912).

Male *O. bidens*-specific expanded genes may be related to biological traits. Male *O. bidens* show secondary sexual characteristics, such as nuptial organs on the surface of the head and extended-anal fins, in the reproductive season. These secondary sexual characteristics are a result of the secretion of male hormones. The KEGG enrichment analysis showed that the expanded genes were overrepresented in the GnRH signaling pathway (Figure 4). GnRH is the primary regulator of luteinizing hormone (LH) and follicle-stimulating hormone synthesis and secretion. The 78 expanded genes in the gonadotropin secretion pathway included LH and the glycoprotein hormone alpha chain (GSUa/CGa). The genes p38MAPK and CaMK (CAMK2) expanded up to eight and seven copies, respectively (Table S6), suggesting an essential role for pituitary gonadotropes secreted from the anterior pituitary gland.

**Figure 5:**
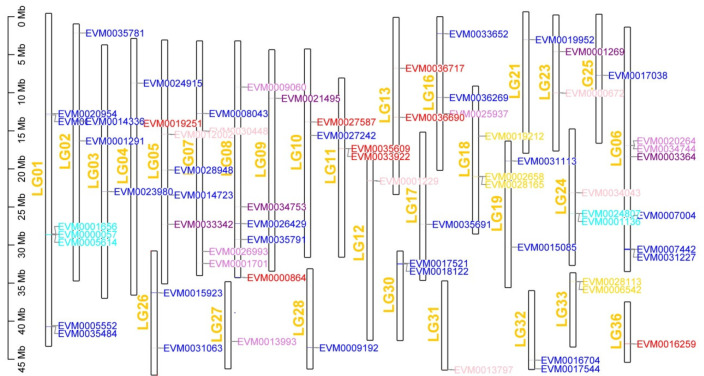
Physical distribution of GnRH signaling pathway-expanded genes in male *O. bidens*’ genome. The gene-marked colors indicate gene-expanded number (≥3, see Tables S5 and S6).

To uncover the mechanism of gene expansion, we examined the distribution of the expanded genes in the chromosomes of male *O. bidens*. The 78 expanded genes were dispersed in 29 chromosomes (Figure 5), accounting for 76.32% (29/38) of the whole chromosomes of male *O. bidens*. The top three chromosomes with expanded gene numbers were LG01 (seven genes), LG06 (six genes), and LG08 (five genes). A few expanded genes were clustered in one chromosome; for example, protein kinase C (PKC: PRKCB) with three genes was clustered in LG01 (EVM0000057, EVM0001656, EVM0005614), whereas part of PKC (PRKCB) was coupled in LG24 (EVM0001136, EVM0024807). This is true for PLA2 (EVM0020264 and EVM0034744) in LG06, and EVM0001701 and EVM0026993 in LG07, indicating that a piece of chromosome breaks off, resulting in gene expansions [22].

### 3.5. Chromosome Breakage and Reunion Verified by Synteny Comparison

**Figure 6:**
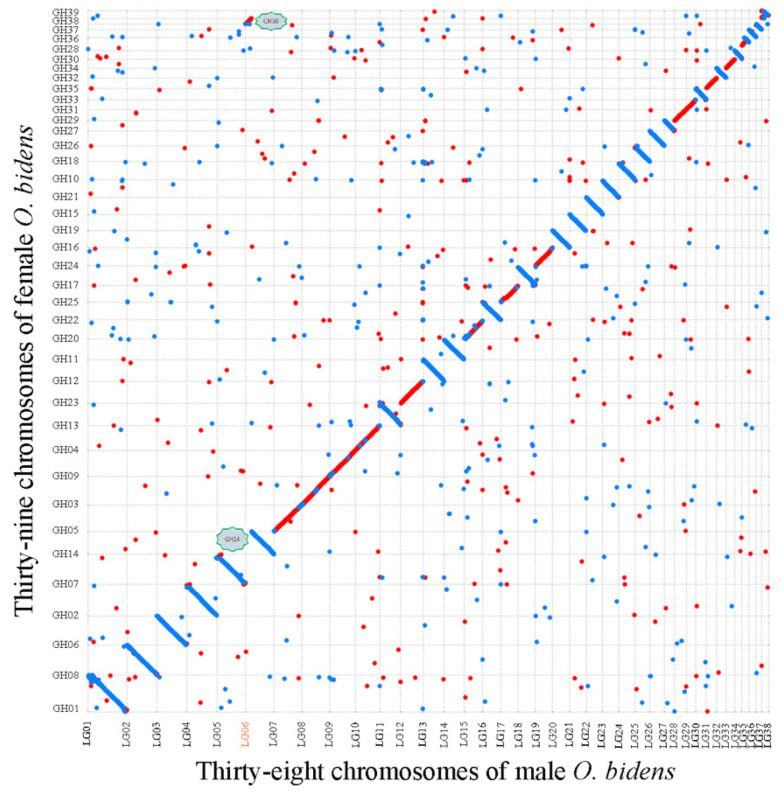
Genomic comparisons between female and male *O. bidens*. Genomic comparisons of whole genome sequences directly, and the majority of female and male *O. bidens* chromosomes exhibited 1:1 correspondence except for chromosomes LG06 in male and GH14 and GH38 in female.

**Figure 7:**
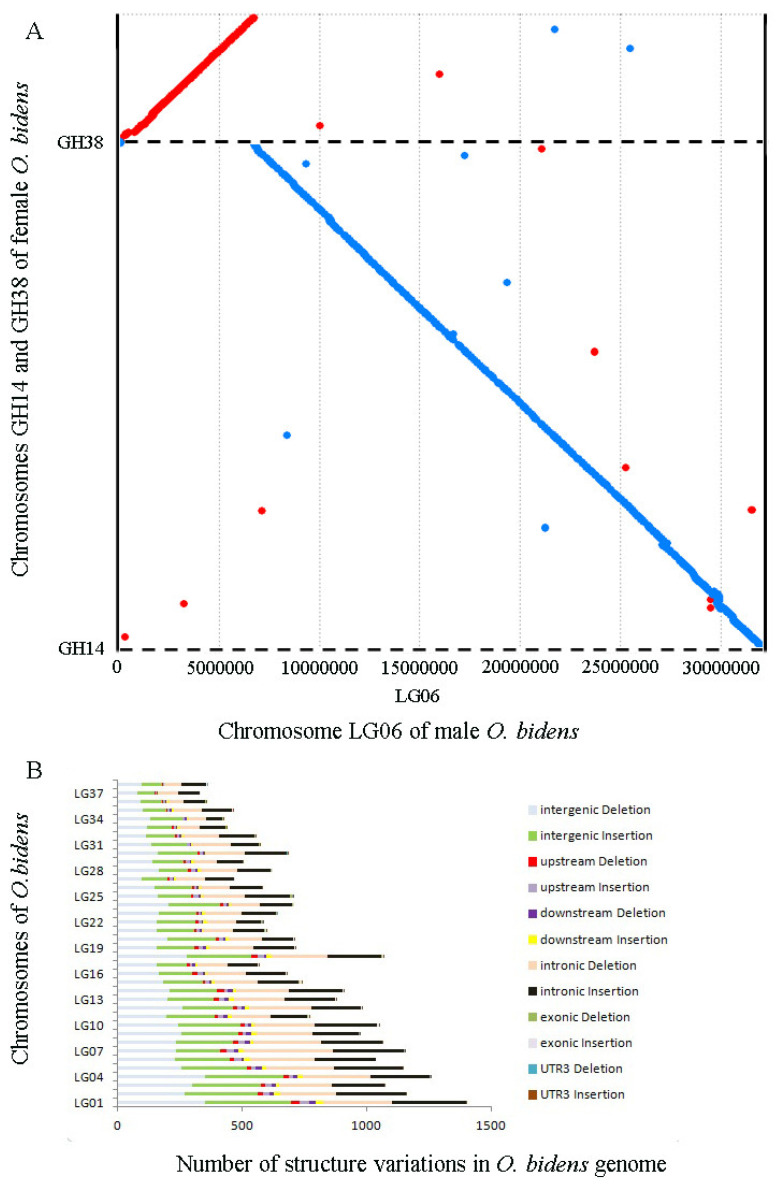
Detail chromosome comparisons between LG06, GH14, and GH38, and structure variation between female and male *O. bidens* genomes. (**A**) The chromosome LG06 of male *O. bidens* genome showed significant synteny with GH14 and GH38 of female *O. bidens* genome. (**B**) Statistics of the structural variations in various regions of the male genome by alignment with female *O. bidens* genome.

To verify the hypothesis of chromosome breakage and reunion, comparative chromosome analysis was performed using MUMmer4 to identify genome-scale syntenic regions between female and male *O. bidens*. The genome sequences corresponding to their chromosomes were highly consistent (Figure 6). Similarly, the chromosomal sequences of female GH14/GH38 exhibited a significant identity with the male LG06 using MUMmer4 with a minimum match length of 1000 bp and 85% of filter (Figure 7A). The female GH14/GH38 chromosomal sequence alignment to the male LG06 showed identities of 97.93% and 97.72%, respectively, suggesting that the female GH14 and GH38 fused to form the male LG06. Indeed, structural variations were identified by performing sequence comparisons, and we found that, in the male LG06, the deletions included 232 in the intergenic region, 20 in the upstream region, 12 in the downstream area, and 259 in the intronic region. The insertion sites included 218 in the intergenic region, 26 in the upstream region, 23 in the downstream region, and 248 in the intronic area. Finally, the duplication, inversion, and translocation sites lacked a homolog to the female GH14/GH38 (Figure 7B). The deletions and insertions that occurred in the male LG06 were lower. They accounted for 3.4% (1038/30,202) of the entire variable sites in all-male *O. bidens* chromosomes, suggesting that the male LG06 resulted from the fusion of the female GH14 and GH38 rather than from massive rearrangements.

According to the annotated gene sets in the male *O. bidens* genome, using homology searching of the female *O. bidens* genome, we identified seven male-specific genes in the male LG06 chromosome, including four uncharacterized protein genes; two genes involved in replication, recombination, and repair functions (EVM0022038, EVM0026331); and one gene annotated as DNA polymerase epsilon subunit 2 (EVM0017949), engaged in DNA-dependent DNA replication in biological processes.

**Figure 8:**
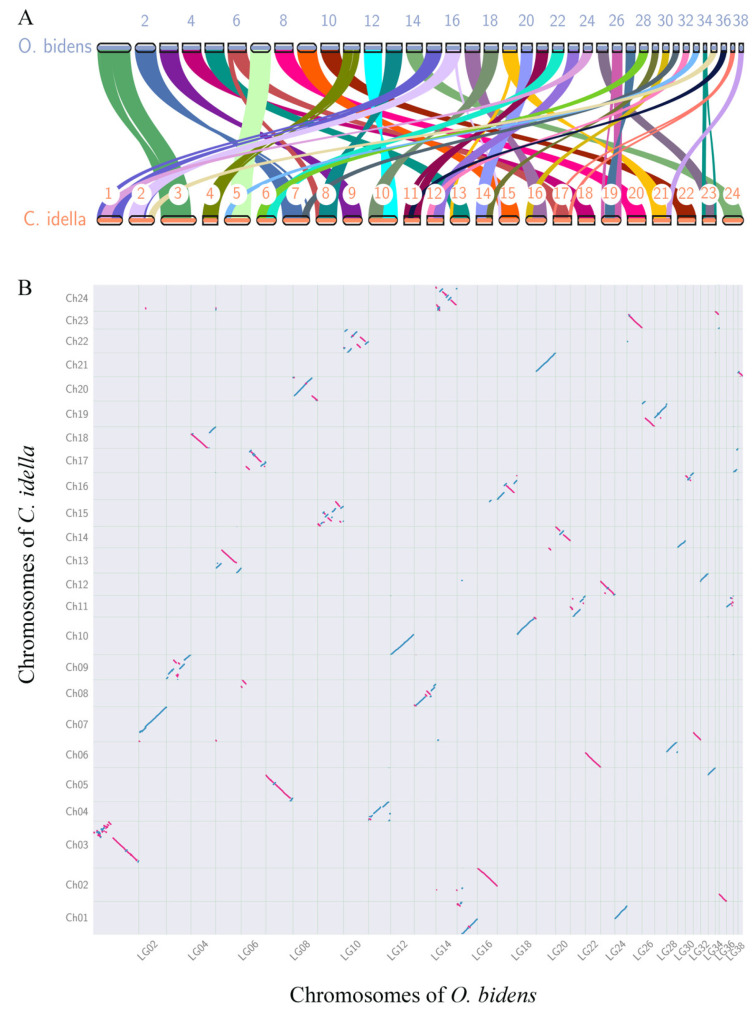
Chromosome conserved synteny analysis between male *O. bidens* and grass carp genome. (**A**) The chromosomes in the two genomes showed multiple chromosomes breaking off in grass carps corresponding to chromosomes in male *O. bidens*. (**B**) Detail inter-genomic conserved blocks with 21,628 gene pairs between grass carp and male *O. bidens* genome.

To reveal the possible mechanism of chromosome number variation by breakage in Cyprinid fish, the chromosomes of male *O. bidens* were aligned to a single chromosome of *C. idella*, exhibiting significant inter-chromosomal conservation (Figure 8A). *C. idella* showed a high homology and 269 synteny blocks with 21,628 gene pairs in the genome comparison. Each of the fourteen *C. idella* chromosomes broke into two chromosomes of the male *O. bidens*, and massive inter-chromosomal rearrangements occurred after the divergence of the two species (Figure 8B). Notably, the fusion of the broken fragments from *C. idella* Ch08 and Ch17 chromosomes gave rise to the LG06 chromosome of male *O. bidens*, indicating that chromosome breakages help to increase their number in fish chromosomal evolution.

## 4. Discussion

### 4.1. Quality of the Assembled Genome at the Chromosome Level

*O. bidens* is a vital aquaculture fish in East Asia that shows sexual dimorphism in growth and body size, with male fish exhibiting faster and greater growth than females [9]. This species could be exploited as a monosexual fish in aquaculture and used as a model to address gene function in sexual dimorphism. In combination with the released genome sequences of female *O. bidens* [10], the male *O. bidens* genome assembled at the chromosome level presented herein will provide valuable genomic resources for gaining critical insight into the regulatory mechanism underlying sexual dimorphism, revealing the sexual complexity and genetic diversity of the species genome.

The quality of the male *O. bidens* genome assembly was evaluated based on continuity and completeness. The male *O. bidens* final contig assembly was 992.9 Mb with a contig N50 length of 5.2 Mb. In the released female *O. bidens* genome [10], the contig assembly was 818.75 Mb with an N50 length of 4.71 Mb. Compared with the female *O. bidens* genome assembly, the N50 length of the current male genome contig was much longer. The male genome was larger than the female’s, suggesting more annotated genes in the male genome (30,922) than in the female genome (23,992 annotated genes). The number of annotated genes in the male genome (30,922) was similar to that of grass carp (30,342), which is closely related to *O. bidens* [10,58], but was higher than that in the female *O. bidens* genome based on the *D. rerio* NCBI database (22,835, data not shown). This may be due to the heterozygous rate of the male genome (0.58%) being higher than that of the female genome (0.36%) in *O. bidens*. Heterozygous describes the presence of two different alleles for the same gene, and higher genome heterozygosity leads to a more diverse gene composition and more genes encoding secreted proteins in fungi [60]. In addition, there are usually differences in the annotated gene numbers between female and male genomes in fishes (e.g., 2.4% for bighead carp [18,61] and 3.5% for *O. fasciatus* [23]). Furthermore, the large contig N50 length is conducive to gene annotation, and when contig N50 ranged from 5.2 Mb [15] to 19.3 Mb [58], the total genome number of female grass carp can reach 11.3% of the annotated genes. The male *O. bidens* genome using Hi-C chromatin interaction maps was 886.81 Mb and anchored to 38 chromosomes ranging from 6.51 Mb to 47.68 Mb, consistent with the 2n = 76 karyotypes. The female *O. bidens* genome is 818.78 Mb and anchored to 39 chromosomes ranging from 6.77 Mb to 42.84 Mb, suggesting that *O. bidens* harbors different chromosomes in sexual individuals and that males have the longest chromosome (LG01, ~47.68 Mb). This study provides comparative and evolutionary studies of male and female *O. bidens* using genome sequences and further identifies the genes and regulatory elements related to sexual dimorphism in growth.

*O. bidens* genomes were larger than many reported Cyprinid genomes (Table S1). Transposons (RNA and DNA types) and the SSR content contribute to genome size. Larger genomes exhibit richer transposons in comparison with teleost fish genomes with annotated transposons [27], such as the 1.37 Gb zebrafish genome with a repeat content of 52.2% [62]. The male *O. bidens* genome showed repeat elements accounting for 43.23% of the 992.9 Mb genome. Using BUSCO methods to evaluate completeness, we found that the male *O. bidens* assembled genome contained 97.5% of the complete sequences (Table S3). The high continuity and completeness of the male *O. bidens* genome will lay a solid foundation for further studies on population genetics, sex-determining mechanisms, sexual dimorphism, growth regulation, and aquaculture breeding [61,63].

### 4.2. Male Sexual Dimorphism-Related GnRH Signaling Pathway

GnRH is secreted from the hypothalamus and plays a key role in the control of vertebrate reproductive functions by binding to its specific membrane receptor, GnRHR, triggering the synthesis and release of pituitary gonadotropins, including follicle-stimulating hormone, LH, and CG, to induce gonads that produce sex steroids for gametogenesis [64,65]. In the hypothalamus–pituitary–gonadal axis, the GnRH signaling cascade control process is essential for maintaining gonadotropin synthesis and release. It plays an important role in developing secondary sex characteristics [66].

Almost all gene families in the GnRH signaling pathway have been identified in the male *O. bidens* genome, and multiple expanded genes are present in the node steps of this pathway. First, GnRHR transmits its signal to activate phospholipase C by coupling channel proteins, such as guanine nucleotide-binding protein subunits Gs and Gq/11. The subtypes of G proteins and their receptors represent the components of a highly versatile signal transduction system, centrally involved in hormone release and actions [67]. Receptor-G protein coupling requires diacylglycerol (DAG) and inositol 1,4,5-trisphosphate (IP3) after ligand-dependent receptor activation, and the G protein occasionally occurs in a cell-specific manner [68,69]. The intracellular PKC pathway supports the primary mechanism for hormone release and action activated via DAG by releasing intracellular calcium and activating the calcium-signaling pathway stimulated via IP3 [70].

In the present study, out of 138 genes involved in the GnRH signaling pathway in male *O. bidens*, 78 showed at least a three-fold increase in essential nodes regulating gonadotropin hormone secretion by pituitary gonadotrope cells. The top three gene families were CaMKII, p38MAPK, and cytosolic phospholipase A2 (PLA2). It has been reported that the Ca^2+^ increase is fast and transient upon the binding of most hormone receptors and that CaMKII, which is a ubiquitous serine/threonine protein kinase, is enriched in the IP3 pathway and activated by Ca^2+^ signals and calmodulin (CaM) to phosphorylate diverse substrates involved in metabolism and hormone release [71]. Synthesis and secretion of LH are influenced by cytokines transported to the pituitary gland through the blood, and act on endocrine pituitary cells. The p38MAPK and PLA2 genes are enriched in the PKC pathway, and when p38 MAPK is inhibited by a small-molecule inhibitor, the LH expression decreases at the mRNA level [18]. PLA2 is a large family of calcium-dependent phospholipases with the ability to directly interact with G-proteins and kinases [72], resulting in short sequestration in activating regulated LH secretion [73]. Production of LH induces growth hormone (GH) and regulates GH secretion and synthesis [74,75,76]. GnRHs have been demonstrated to up-regulate GH mRNA expression in goldfish [77], grass carp [78], and masu salmon [79]. The physiological roles of GnRH are mediated by GnRH receptor (GnRHRs) on target tissues, which include GnRHR I, GnRHR IIa, and GnRHR IIb. GnRHR IIb (designated as GnRHR2) expressed in somatotropes was bound by GnRHR IIa (designated as GnRH1) to stimulate GH release in teleosts. The ricefield eel *Monopterus albus*, a protogynous hermaphroditic fish, showed sex reversal from female to male and a larger male body. Along with development, mRNA products of GnRHR2 and GH were co-increased, resulting in the male growing faster than the female [75]. GnRHR1 (EVM0031063) and GnRHR2 (EVM0001291) have been identified in the male *O. bidens* genome, indicating that the expanded genes in the GnRH signaling pathway play an important role in sexual dimorphism in the growth of male *O. bidens*.

### 4.3. Mechanism Underlying O. bidens Chromosome Number Variety

During vertebrate evolution, the entire genome underwent two rounds (2R) of duplication, and fish-specific genome duplication, namely the third round of whole-genome duplication (3R) approximately 320 million years ago, subsequently developing into new diploids [80]. Therefore, most teleosts carry about 50 chromosomes in diploid fish species [81]. The 4R occurred in certain Cyprinidae fish, resulting in palaeopolyploids with 100 chromosomes in their nuclear genome, such as the common carp *C. carpio* and *S. rhinocerous* in the tetraploidized chromosome [13,80,82,83]. Multiple rounds of whole genome duplication produce redundant genes that provide important genetic material for phenotypic complexity, which would potentially benefit an organism in its molecular functions. Interestingly, the Cyprinid fish *O. bidens* is an exception, and different populations show various chromosome numbers (between 74 and 78) [22]. The chromosomal evolution of Cyprinid remains a subject of debate.

Recently, the released genome of female *O. bidens* has provided an opportunity to investigate the mechanism underlying chromosome changes [10]. Female *O. bidens* carry 39 (2n = 78) chromosomes. In comparison, male *O. bidens* were shown to carry 38 (2n = 76) chromosomes in the present study, suggesting a difference in the regulation of sexual dimorphism in hook snout carp at the chromosomal level. The male *O. bidens* LG06 chromosome was formed from a reunion of female GH14 and GH38 chromosomes and the comparison showed more than 97% sequence similarity between males and females. It has been verified that *O. fasciatus*, which possesses 2n = 47 in males and 2n = 48 in females, carries a large metacentric Y chromosome [84]. Different karyotypes in *O. punctatus* are responsible for the genome size discrepancy between male and female individuals [85]. This suggests that *O. bidens* has a sex-dependent system. In the male *O. bidens* genome, GnRH-induced hormone release-related gene families underwent expansion, and up to seven expanded genes were distributed in the LG06 chromosome. Therefore, it is reasonable to regard LG06 as a sexually dimorphic chromosome in growth, and the related molecular mechanisms are worth investigating in future studies.

The hook snout carp displayed an atypical karyotype of 3R. Our genome assembly for male *O. bidens* led to 38 chromosomes, directly corresponding to male karyotypes (2n = 76). Although different populations carry different chromosome numbers, the fundamental arm number of their karyotypes is 86 [22,86]. The leuciscin fishes, including *Ochetobius elongatus* (Kner), *Luciobrama macrocephalus* (Lacepède), *Elopichthys bambusa* (Richardson), *Squaliobarbus curriculus* (Richardson), and *C. idellus*, carry 2n = 48 chromosomes, with fundamental arm numbers of 74, 82, 82, 90, and 92, respectively [86]. Hook snout carp have similar fundamental arm numbers to Leuciscin fishes, suggesting that the 76 chromosomes of male *O. bidens* resulted from the 2n = 48 chromosomes of Leuciscin fishes by breakage at centromere sites. Considering that *C. idellus* is regarded as a primitive species in Leuciscin fish species evolution [58], the synteny-based chromosome comparison between male *O. bidens* and *C. idellus* showed that out of the 24 chromosomes in *C. idellus*, one breakage into two chromosomes occurred in 12 chromosomes, providing evidence of added chromosome number via breakage. Our results could serve as a framework for studies on sex-determining mechanisms and genome evolution in fish.

## 5. Conclusions

This is the first study to present a high-quality and -continuity chromosome-level genome assembly of male *O. bidens* using nanopore long-read sequencing, achieving a total genome size of 992.9 Mb with a contig N50 of 5.2 Mb. Using Hi-C sequencing technology, 89.31% of the genome sequences were anchored to 38 chromosomes with scaffold N50 of 19.44 Mb corresponding to male karyotypes. A total of 30,922 functional genes were annotated using various databases, and 43.23% of the male genome was identified as repetitive elements. Over 97% of the BUSCO genes were completely identified in the male *O. bidens* genome, indicating a complete genome assembly. Therefore, the male *O. bidens* genome can serve as genetic material for the investigation of the role of male-specific sexual dimorphism in growth. We identified many expanded gene families involved in the synthesis and secretion of hormones in the GnRH signaling pathway. The relevant genes act on growth hormones, regarded as the dominant regulators of sexual dimorphism in growth. These GnRH signaling-related expanded genes were distributed in multiple male *O. bidens* chromosomes. The gene number was between one and seven, and the top three were chromosomes LG01, LG06, and LG08. Compared to the released female *O. bidens* genome, we found that the male *O. bidens* LG06 resulted from a reunion of female *O. bidens* chromosomes GH14 and GH38, carrying male-specific genes involved in DNA replication, recombination, and repair. Further study of LG06 revealed that male *O. bidens* LG06 originated from the ancient fusion of large fragments from grass carp chromosomes breaking off by genome synteny comparison. This study provides valuable genomic resources for subsequent research on the growth and development, sexual-controlled mechanisms, sex-determination systems, and molecular breeding of *O. bidens*.

## Figures and Tables

**Table 1:** Repetitive sequences in the *O. bidens* genome.

Types	Number	Length (bp)	Rate (%)
Class I	1,139,895	232,332,806	23.34
DIRS	105,253	29,919,018	3.01
LARD	655,447	119,102,747	11.99
LINE	223,903	52,367,782	5.27
Copia	7429	2,540,946	0.26
Gypsy	61,709	19,444,768	1.96
PLE	24,473	8,156,447	0.82
SINE	1725	316,380	0.03
Unknown	47,754	15,326,877	1.54
Class II	785,886	137,824,754	12.59
Crypton	11,798	1,634,260	0.16
Helitron	35,267	9,129,144	0.92
Maverick	4578	567,260	0.06
TIR	601,449	113,701,347	11.45
Unknown	132,794	17,751,657	1.79
SSR	7405	5,817,413	0.59
Unknown	145,781	33,606,673	3.38
Total	2,078,967	357,309,255	43.23

**Table 2:** Overview of predicted non-coding RNAs.

ncRNA	Number in of Loci	Average Length (bp)	Family
tRNA	3280	74.37	25
rRNA	5616	149.31	4
miRNA	450	87.52	88
snRNA	449	151.74	7
snoRNA	339	185.61	4

## Data Availability

The BioProject from genomic sequencing was assigned CNGB (China National GeneBank, https://ngdc.cncb.ac.cn/, accessed on 18 August 2022). The project accession number was PRJCA010154. The assembly accession number was GWHBJYU00000000. The sequences and genome annotation files were deposited in the CNGB under CRA007262 for genome using Nanopore long reads data, CRA007264 for the assembled genome using Hi-C sequencing data, CRA007266 for Illumina short reads raw data, and CRA007267 for RNA-Seq raw data.

## Supplementary Materials

The following supporting information can be downloaded at: https://www.mdpi.com/article/10.3390/biology11101500/s1, Figure S1: K-mer distribution of the *O. bidens* genome; Figure S2: Statistics of the expanded and contracted gene families in *O. bidens* genome by GO annotation; Figure S3: Top ten statistics of the expanded and contracted gene families in *O. bidens* genome by KEGG annotation; Table S1: Information of species for phylogenetic analysis; Table S2: Summary of data used for the assembled male *Opsariichthys bidens* genome; Table S3: Integrity statistics of the assembled genome of *Opsariichthys bidens*; Table S4: Statistics of gene annotation of *Opsariichthys bidens*; Table S5: Integrated_Function.annotation; Table S6: KEGG pathway list.
