# Supplementary material for: Chromosome-Level Assembly of Male Opsariichthys bidens Genome Provides Insights into the Regulation of the GnRH Signaling Pathway and Genome Evolution
Source: Biology (Basel). 2022 Oct 13;11(10):1500. doi: 10.3390/biology11101500 (PMC9598921; doi:10.3390/biology11101500)
Supplement: Supplementary file 1 [file biology-11-01500-s001.zip › Figure S1-S3.pdf]

Supplementary Figures

Figure S1-S3

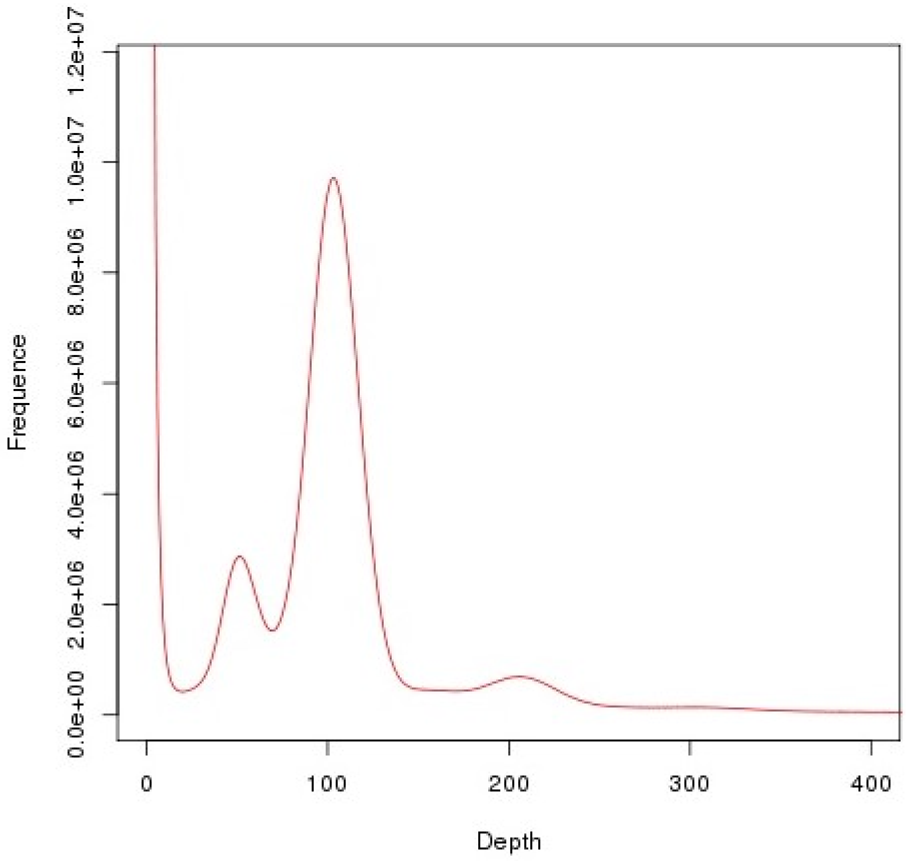

Figure S1. K-mer distribution of the *O. bidens* genome. After the error-excluded kmers were excluded, 813.1 Mb of the observed kmers based on the 17-kmer peak with depth of 111.3 × coverage; the heterozygous rate of genome was 0.58%.

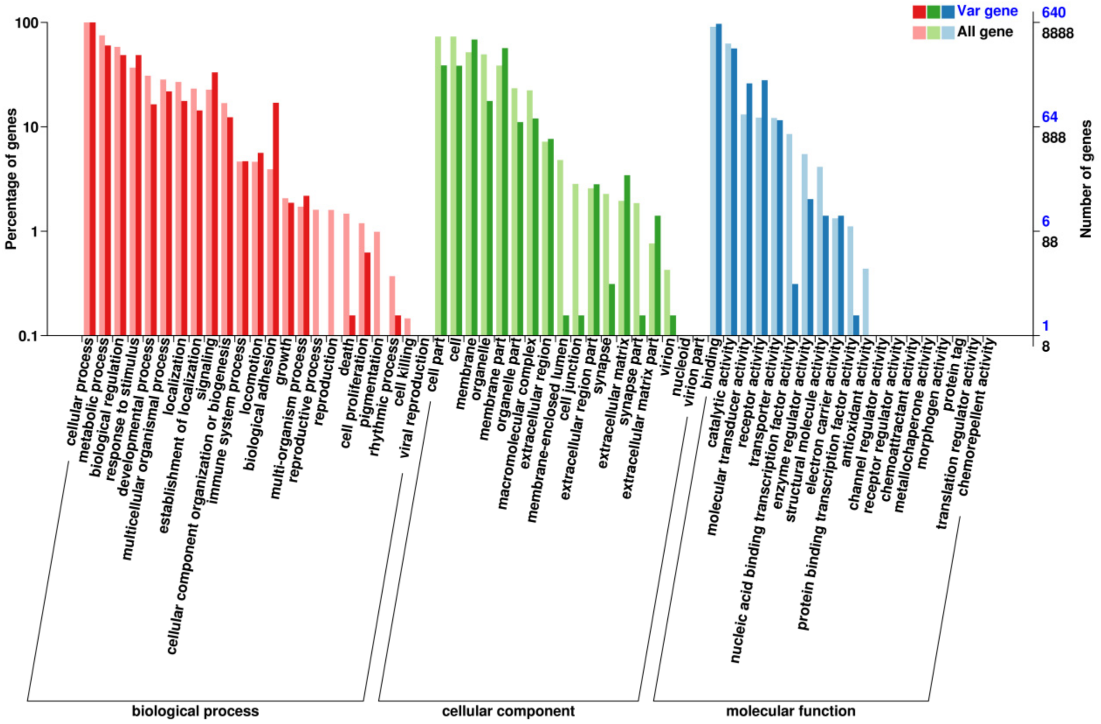

Figure S2. Statistics of the expanded and contracted gene families in *O. bidens* genome by GO annotation

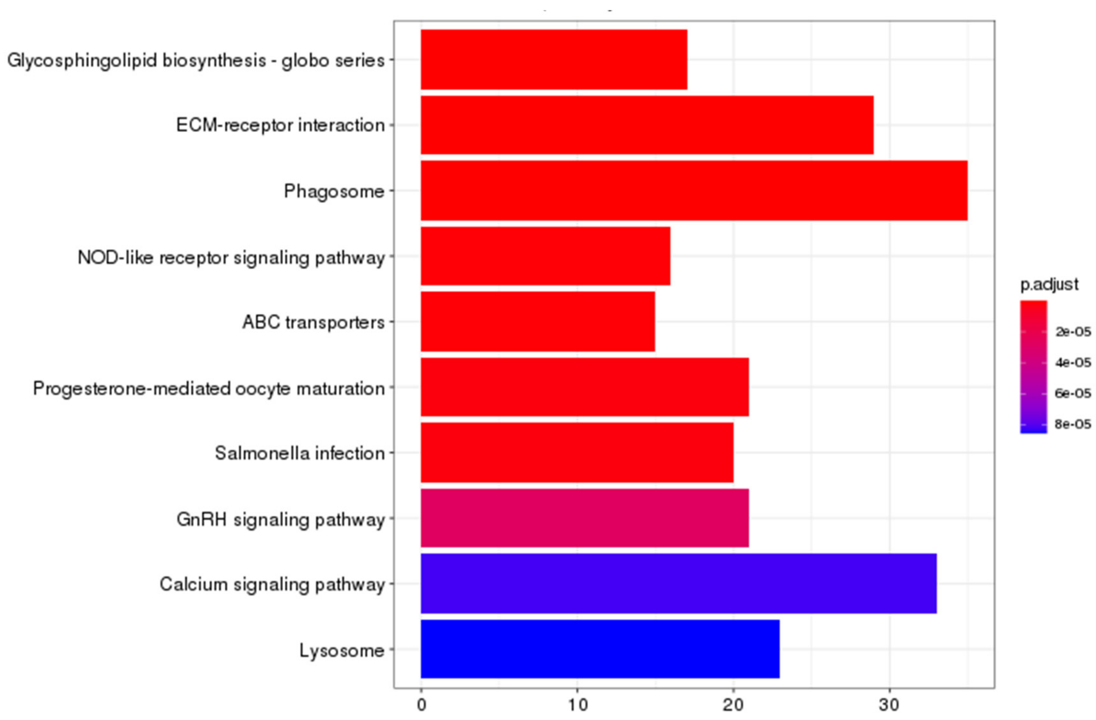

Figure S3. Top ten statistics of the expanded and contracted gene families in *O. bidens* genome by KEGG annotation
