# Supplementary material for: Chromosome-Level Assembly of Male Opsariichthys bidens Genome Provides Insights into the Regulation of the GnRH Signaling Pathway and Genome Evolution
Source: Biology (Basel). 2022 Oct 13;11(10):1500. doi: 10.3390/biology11101500 (PMC9598921; doi:10.3390/biology11101500)
Supplement: Supplementary file 1 [file biology-11-01500-s001.zip › Table S1-S4 O.bedins.pdf]

Table S1-S4

Table S1. Information of species for phylogenetic analysis

Species	Genome accession	Size (Mb)	Assembly Level
Sinocyclocheilus rhinocerous	GCF_001515625.1	1655.79	Scaffold
Anabarilius grahami	GCA_003731715.1	992	Scaffold
Labeo rohita	GCA_022985175.1	1126.54	Chromosome
Danio rerio	GCF_000002035.6	1,373.45	Chromosome
Oryzias latipes	GCF_002234675.1	734.04	Chromosome
Takifugu rubripes	GCF_901000725.2	384.11	Chromosome
Cyprinus carpio	GCF_018340385.1	1,680.12	Chromosome
Gasterosteus aculeatus	GCF_016920845.1	471.88	Chromosome

Table S2. **Summary of data used for the assembled male *Opsariichthys bidens* genome.**

Strategy	Method	Insert size	Raw data (Gb)	Genome size (Mb)	Coverage (fold)*	Contig number	Contig N50 length (bp)	Scaffold number	Scaffold N50 length (bp)
Genome	Illumina NovaSeq	350bp	90.47	813.1	111.3	1,359,694	1,643	1,125,882	2,798
Genome	Nanopore PromethION	27kb	99.7	992.91	122.6	1,373	5,205,634	/	/
Hi-C	Illumina HiSeq 2500	350bp	55.96	886.81	68.8	1,864	2,852,175	1,461	19,439,068

*The coverage was calculated using an estimated genome size of 813.1 Gb.

Table S3. Integrity statistics of the assembled genome of *Opsariichthys bidens*

Methods	Parameters				
**Illumina short reads mapping**	**Total reads**	**Mapped reads**	**Mapped (%)**	**Properly mapped reads**	**Properly mapped (%)**
	381,811,106	379,964,912	99.52	367,328,604	96.91
**CEG database assess**	**Number of 458 CEG present in assembly**	**458 CEGs present in assembly (%)**	**Number of 248 conserved CEGs present**	**248 conserved CEGs present (%)**	
	456	99.56%	246	99.19%	
**BUSCO assess**	**Complete BUSCOs (%)**	**Complete single-copy BUSCOs (%)**	**Complete duplicated BUSCOs (%)**	**Fragmented BUSCOs (%)**	**Missing BUSCOs(%)**
	2521 (97.49%)	2415 (93.39%)	106 (4.10%)	13 (0.50%)	52 (2.01%)

Table S4 Statistics of gene annotation of *Opsariichthys bidens*

Method	Software	Species	Gene number
**De novo**	Genscan	-	40,007
	Augustus	-	58,897
	GlimmerHMM	-	38,190
	GeneID	-	43,381
	SNAP	-	92,095
**Homology-based**	GeMoMa	*Danio rerio*	28,692
		Ctenopharyngodon idellus	34,173
**RNA seq**	TransDecoder	-	38,261
	GeneMarkS-T	-	35,471
	PASA	-	26,480
**Integration**	EVM	-	36,738
